# Disinfectant and antibiotic resistance in *Mycobacterium abscessus* water isolates

**DOI:** 10.1128/spectrum.03374-24

**Published:** 2025-06-10

**Authors:** Kirby Patterson-Fahy, Robyn Carter, Steven L. Taylor, Jianhua Guo, Rachel M. Thomson

**Affiliations:** 1 The University of Queensland, Greenslopes Clinical School, Gallipoli Medical Research, Greenslopes, Queensland, Australia; 2 Microbiome and Host Health Program, South Australia Health and Medical Research Institute, Adelaide, South Australia, Australia; 3 College of Medicine and Public Health, Flinders University 1065, Adelaide, South Australia, Australia; 4 Australian Center for Water and Environmental Biotechnology (ACWEB, formerly AWMC), The University of Queensland, St. Lucia, Queensland, Australia; 5 Department of Thoracic Medicine, The Prince Charles Hospital, Chermside, Queensland, Australia; University of Manitoba, Winnipeg, Manitoba, Canada

**Keywords:** nontuberculous mycobacteria, chlorine, chloramine, antimicrobial resistance, disinfection, drinking water

## Abstract

*Mycobacterium abscessus* has been found in drinking water distribution systems worldwide, and infections have been increasing in frequency. The emergence of dominant circulating clones within clinical settings and during chronic disease has been considered a potential cause of the increasing frequency of disease. However, *M. abscessus* has been thought to be largely environmentally acquired, and how selective pressures in the environment may be influencing *M. abscessus* evolution has not previously been considered. This study aimed to investigate the disinfectant and antibiotic susceptibility of *M. abscessus* isolated from drinking water treated with both chlorine and chloramine in 2007, 2017–2018, and 2021–2022 as well as a laboratory evolution experiment. There was no trend in disinfectant or antibiotic resistance of water isolates over time, although there were significant differences between subspecies and dominant circulating clones. *M. abscessus* isolates were found to be significantly more susceptible to chloramine than chlorine, yet both MICs were greater than the concentrations used in water treatment. The laboratory evolution experiment resulted in a chlorine-resistant phenotype that was not a heritable genetic change. Exposure to chloramine resulted in decreased imipenem susceptibility without a change in chloramine susceptibility. Overall, the results of this study show that *M. abscessus* is highly resistant to disinfection, and exposure to disinfectants within drinking water distribution systems could influence antimicrobial susceptibility.

**IMPORTANCE:**

*Mycobacterium abscessus* causes significant disease and is present in drinking water distribution systems where it is exposed to chlorine and chloramine. In this study, *M. abscessus* drinking water isolates were highly resistant to both chlorine and chloramine, with significant differences within the *M. abscessus* group. A laboratory evolution experiment induced chlorine resistance, and exposure to chloramine resulted in decreased imipenem susceptibility. These results suggest that exposure to disinfectants within drinking water distribution systems could result in increased disinfectant and antibiotic resistance, potentially contributing to the increasing frequency of disease.

## INTRODUCTION

*Mycobacterium abscessus* (Mabs) is an opportunistic pathogen most commonly causing pulmonary disease in people with structural lung disease. Mabs pulmonary disease causes significant morbidity and mortality, with treatment requiring greater than a year of antibiotics and success rates as low as 33% (1). This is in part due to Mabs high intrinsic and acquired antibiotic resistance (2). Mabs comprises three subspecies: *M. abscessus* subsp. *abscessus* (Maa), subsp. *bolletii* (Mab), and subsp. *massiliense* (Mam), differentiation of which is clinically significant due to differences in antibiotic resistance and treatment outcomes (1). A full-length *erm(41*) gene is present in Maa and Mab, which confers inducible macrolide resistance (3). A T28C polymorphism in *erm(41*) results in macrolide susceptibility, yet resistance is considered the wild type (4). Mam has a truncated non-functional version of the *erm(41*) gene, which is thought to contribute to its more favorable clinical outcomes (1). Mabs can also acquire constitutive resistance to macrolides and amikacin with point mutations in the 23S and 16S rRNA genes, respectively (5, 6). Macrolides, amikacin, and imipenem are the only antibiotics whose use has been associated with treatment success (1), while resistance to macrolides is associated with treatment failure (7).

The prevalence of Mabs and other non-tuberculous mycobacterial (NTM) pulmonary disease has been increasing in Australia (8) as well as in other parts of the world (9). Recent identification and investigation of dominant circulating clones (DCCs) have suggested that the evolution of increased transmissibility, antibiotic resistance, and virulence in clinical isolates is responsible for the increased frequency of pulmonary disease (10). DCCs are thought to have emerged through gradual adaptation during chronic pulmonary infections and indirect person-to-person transmission via global transmission networks (10, 11). DCCs have been identified in 74% of Mabs pulmonary disease cases in people with cystic fibrosis (12), as well as in a high proportion in drinking water (13).

Mabs pulmonary disease has been thought to be largely environmentally acquired, and the frequency of isolation from drinking water has also been increasing (14). For example, Mabs strains have been detected in drinking water distribution systems (DWDS) in Australia (15) and around the world (16). Water treatment is considered an important variable in DWDS NTM isolation, with increased distance from water treatment associated with increased isolation of NTM (17, 18). Disinfection in DWDS is commonly performed using chlorine and chloramine, which, while effective for most water-borne pathogens, has been shown to be minimally effective for Mabs (19). It is even thought that chlorine and chloramine disinfection facilitate a permissive niche for Mabs to proliferate as faster-growing competition is reduced (20).

The cumulative impact of disinfectants on Mabs genotypic and phenotypic characteristics is poorly understood. In particular, for other microorganisms, low levels of disinfectants have been shown to select for more disinfectant resistance (21), with chlorine-resistant bacteria also more antibiotic resistant (22), and antibiotic resistance genes more prevalent in DWDS after chlorination (23). This is thought to be due to the induction of stress responses in bacteria that result in increased DNA mutation rate and decreased cell wall permeability that lead to antibiotic resistance (24, 25). We, therefore, hypothesized that long-term exposure to disinfectants could co-select antibiotic resistance in Mabs via mutation.

The objective of this study was to investigate how antibiotic and disinfectant susceptibility of Mabs isolated from drinking water may be contributing to the increased frequency of isolation. To this end, we examined Mabs isolated from Brisbane’s DWDS in 2007, 2017–2018, and 2021–2022. Specifically, the present study aimed to investigate (i) if antibiotic susceptibility of the isolates from different time points differed, (ii) if chlorine and chloramine susceptibility of Mabs isolates increased over time, (iii) if exposure to sub-inhibitory concentrations of disinfectant in a laboratory evolution experiment would result in increased resistance to the disinfectants, and (iv) if exposure to sub-inhibitory concentrations would result in antibiotic resistance and/or any genetic changes.

## RESULTS

### Antimicrobial susceptibility testing of *M. abscessus* water isolates

Across the three Mabs isolates from 2007, four isolates from 2017/2018, and five isolates from 2021/2022, there was no pattern of increased antimicrobial resistance over time (Table 1). There were four 2021–2022 isolates that had low MICs for minocycline, with three of the four also having low MICs for doxycycline, where all other isolates were resistant. All water isolates were susceptible to amikacin and clarithromycin.

**TABLE 1:** Antibiotic susceptibility of *M. abscessus* isolated from Brisbane’s municipal water supply in 2007, 2017–2018, and 2021–2022^*b*^^,^^*c*^

Year	Subspecies	MIC (mg/L)									
		DCC	LZD	IMI	FOX	AMI	DOX	MIN^*a*^	TGC^*a*^	CLA (D3)	CLA (D14)
2007	*abscessus*	5	8	8	32	16	>16	>8	0.25	0.12	1
2007	*abscessus*	5	32	16	32	8	>16	>8	0.5	0.25	2
2007	*abscessus*	5	4	8	16	4	>16	>8	0.12	0.12	0.5
2017	*massiliense*	3	8	8	32	16	>16	>8	0.5	0.25	1
2018	*abscessus*	5	16	8	16	8	>16	>8	0.5	0.25	1
2017	*abscessus*	5	32	8	32	4	>16	>8	0.5	0.25	1
2017	*abscessus*	5	16	8	32	4	>16	>8	0.5	0.25	1
2021	*massiliense*	N	32	8	32	8	1	<1	0.12	0.12	0.5
2021	*massiliense*	N	16	8	32	8	0.5	<1	0.25	0.12	0.5
2021	*massiliense*	N	2	16	32	4	4	<1	0.5	0.25	0.25
2021	*abscessus*	5	32	16	64	8	>16	>8	0.25	0.25	1
2022	*massiliense*	N	32	16	64	16	16	2	0.5	0.25	1

^*a*^ No interpretive criteria.

^*b*^ MICs shaded gray are resistant according to the Clinical Laboratory Standards Institute interpretative criteria (26).

^*c*^ LZD, linezolid; IMI, imipenem; FOX, cefoxitin; AMI, amikacin; DOX, doxycycline; MIN, minocycline; CLA (D3), clarithromycin at day 3; CLA (D14), clarithromycin at day 14 (inducible resistance); *N*, non-DCC.

**Fig 1:**
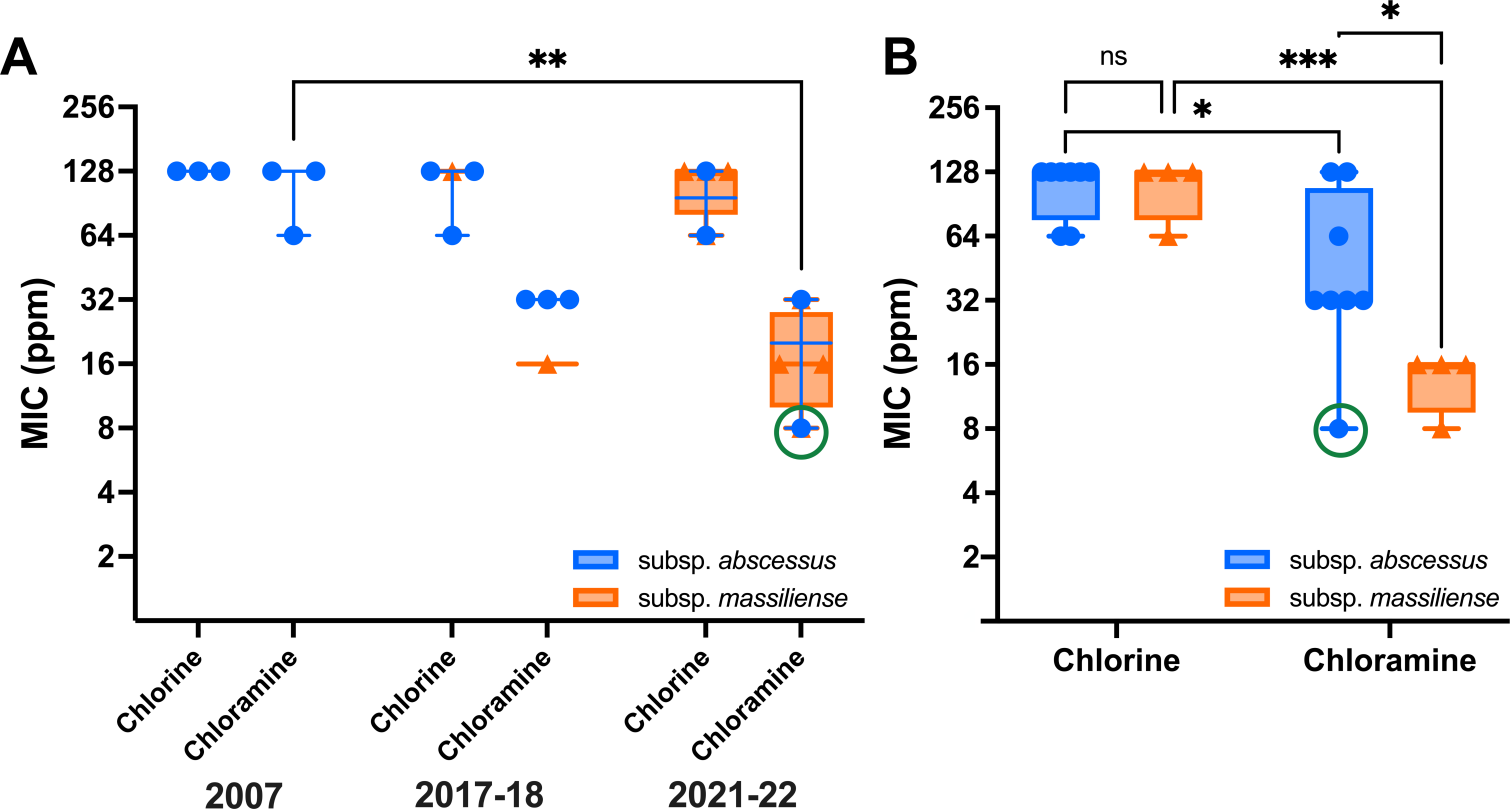
Chlorine and chloramine susceptibility of *M. abscessus* isolated from Brisbane’s municipal water in 2007, 2017–2018, and 2021–2022. (**A**) Susceptibility over time. (**B**) Subspecies difference in susceptibility. The circled isolate is a DCC1, where all other Maa isolates were DCC5. * *P* < 0.05, **P < 0.01, and ***P < 0.001.

There was also no trend in the MICs for chlorine and chloramine across the three time points (Fig. 1A). There was no significant difference in chlorine MICs between Maa and Mam. For Maa, the chloramine MICs were statistically significantly lower for the isolates from 2021 to 2022 compared to the 2007 isolates (*P* = 0.0012). The single isolate from 2021 to 2022 (circled) was a DCC1 isolate, whereas all other Maa isolates were DCC5 isolates. Excluding the DCC1 isolate and considering just the DCC5 Maa isolates, there was no significant difference in the chloramine MIC between 2007 and 2021–2022 for Maa.

Both Maa and Mam were significantly more susceptible to chloramine than chlorine with lower MICs (*P* = 0.0181 and *P* < 0.0001, respectively; Fig. 1B). Mam was significantly more susceptible than Maa to chloramine (*P* = 0.0325), where there was no subspecies difference for chlorine. The Maa DCC5 isolates had significantly higher MICs compared with the other isolates (*P* = 0.0002).

### Impact of prolonged disinfectant exposure on a *M. abscessus* growth and resistance

**Fig 2:**
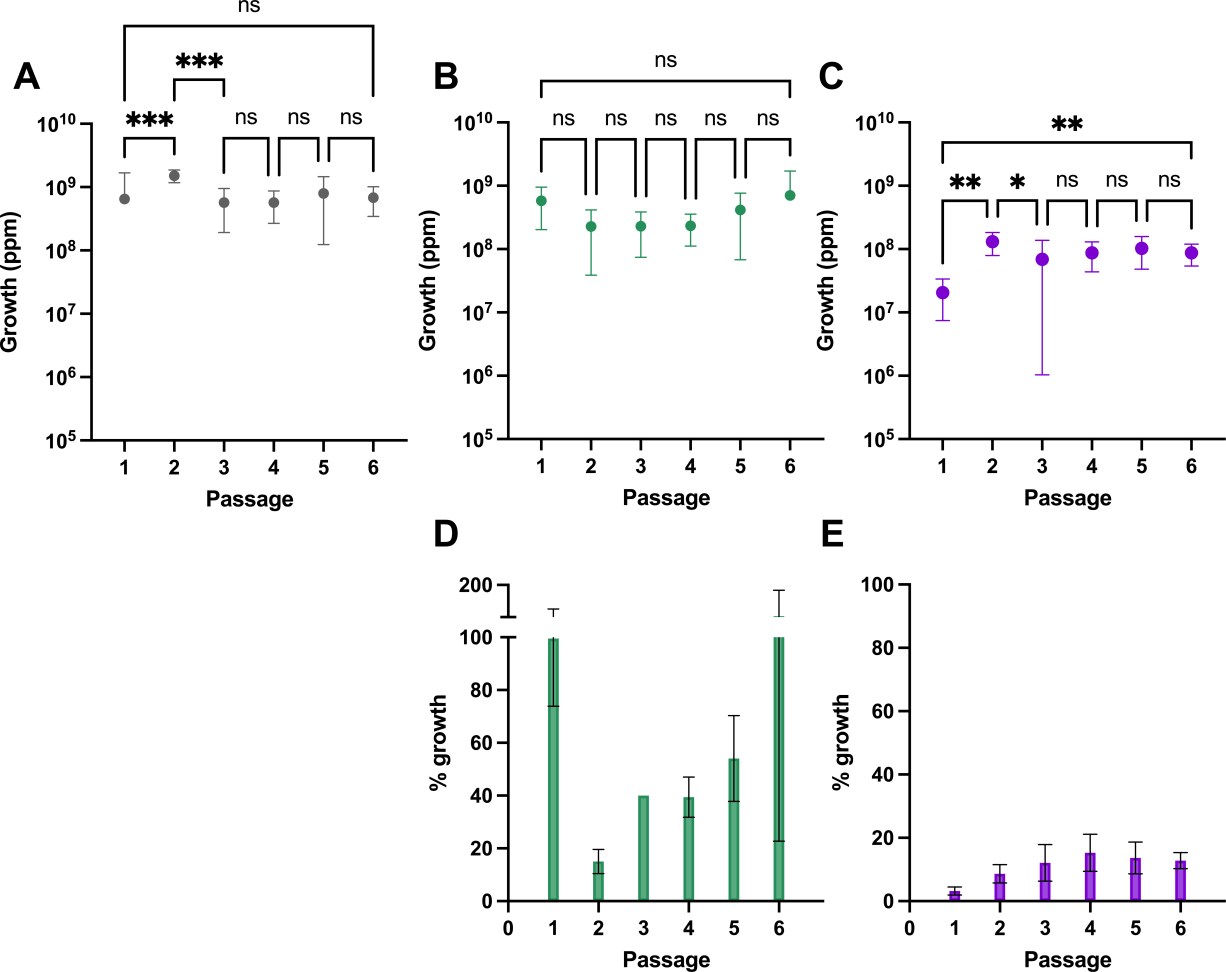
Growth of chlorine and chloramine evolved lines across six passages of the evolution experiment. The calculated CFU per milliliter from colony counts across two dilutions in duplicate for the biological triplicates of each condition presented as the mean and SD. (**A**) Control passaged in broth. (**B**) Chlorine passaged lines. (**C**) Chloramine passaged lines. Percentage growth was calculated as the mean CFU per milliliter divided by the mean CFU per milliliter of the control lines at the same passage. (**D**) Chlorine passaged lines. (**E**) Chloramine passaged lines. *P < 0.05, **P < 0.01, and ***P < 0.001.

A single Maa isolate from 2007 (belonging to DCC5) was selected to assess the impact of repeated exposure to either chlorine or chloramine on growth and antimicrobial resistance. For each of the six passages, the CFU per milliliter was enumerated to assess changes in growth. Both control- and chloramine-passaged Mabs showed no change in CFU (Fig. 2A and B). However, when passaged with chlorine, there was a significant increase in CFU per milliliter from passages 1 to 6 (*P* = 0.0021; Fig. 2C). This was mostly driven by a significant increase from passages 1 to 2 (*P* = 0.0008). The percentage growth (growth for each passage under the selective pressure divided by the average CFU per milliliter for the control at that passage) also showed change for the chlorine lines over the course of the evolution experiment (Fig. 2E). There was a gradual increase from passage 1 (mean = 3.23% and SD = 1.27%) to passage 4 (mean = 15.3% and SD = 5.84%), where it was maintained through to passage 6. The percentage growth of the chloramine passaged lines also showed a trend, with a decrease to 15% (SD = 4.56%) of the control at passage 2 before increasing each subsequent passage to equal the control at passage 6 (mean = 103% and SD = 80.6%; Fig. 2D).

**Fig 3:**
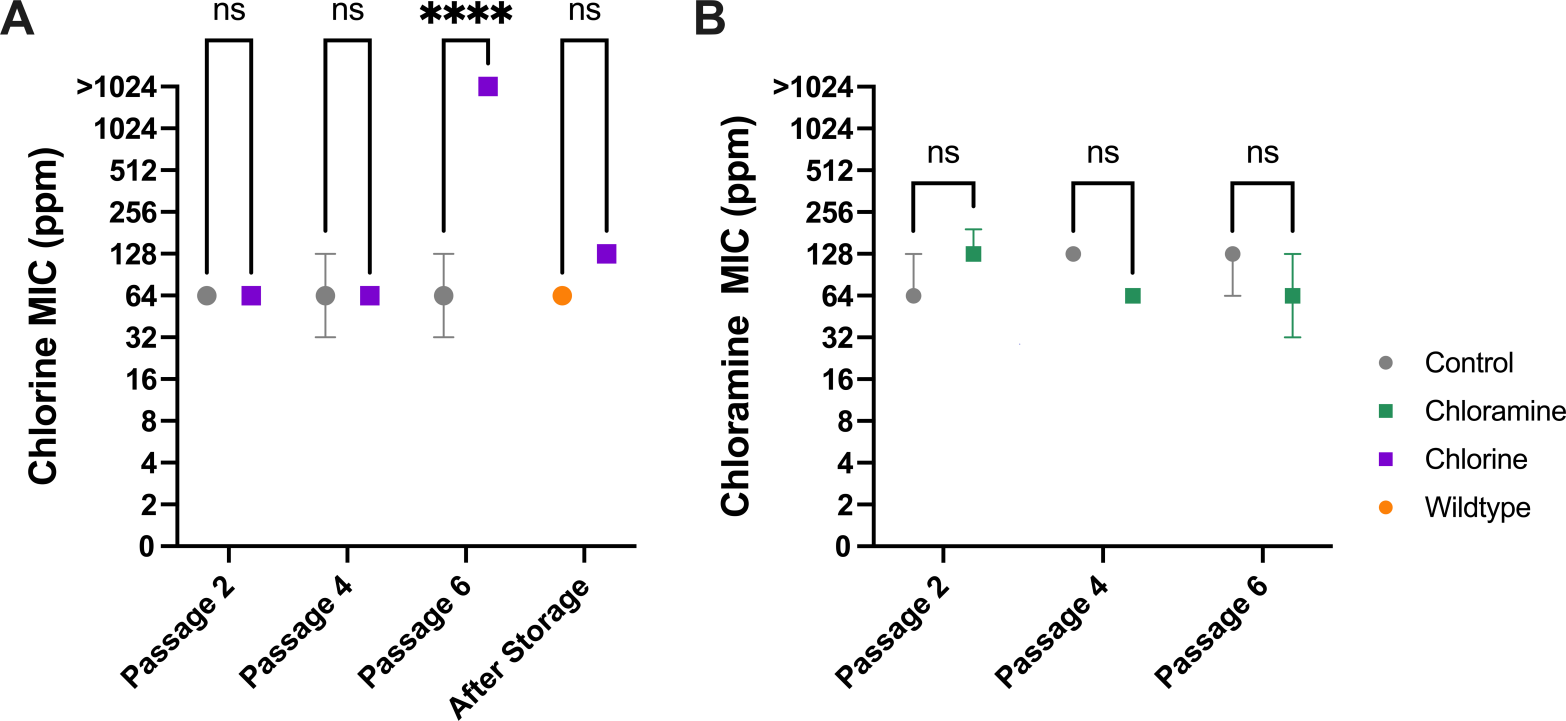
Antimicrobial susceptibility testing results of the chlorine, chloramine, and imipenem passaged lines across passages 2, 4, and 6. Gray represents the control passaged lines susceptibility testing. All data are represented as the median and range of biological triplicate under each condition. (**A**) Chlorine. (**B**) Chloramine. *P < 0.05, **P < 0.01, ***P < 0.001, and ****P < 0.0001.

The corresponding disinfectant MICs were measured after passages 2, 4, and 6. The chlorine-passaged lines had a >16-fold increase in MIC at passage 6, compared to the original isolate (*P* < 0.0001; Fig. 3A). However, after the 6 passages, 6 isolates were stored at −80°C and recultured after approximately 3 months. Chlorine resistance reverted to an MIC similar to the original isolate (Fig. 3A). There was no change in MIC for the chloramine-passaged lines or the control (Fig. 3B).

After passage 6, further antimicrobial susceptibility testing was conducted to detect the potential emergence of multi-drug resistance (Table 2). The chloramine-passaged lines had an increased imipenem MIC (*P* = 0.033), while only one of the lines had an MIC outside the range of the original isolate. The chlorine-passaged Mabs had a significantly decreased amikacin MIC (*P* = 0.0093).

**TABLE 2:** Antibiotic susceptibility at the conclusion of the evolution experiment^*b*^^,^^*c*^

Passaged line	MIC (mg/L)						
	LZD	IMI	FOX	AMI	TGC^*a*^	CLA (D3)	CLA (D14)
Wild type							
	32 (8–32)	8 (4–16)	32 (32–64)	8 (4–16)	0.5 (0.25–0.5)	0.25 (0.12–0.25)	1 (1, 2)
Control							
	32	8	32	8	0.5	0.12	1
	16	8	32	16	1	0.12	1
	16	8	32	4	0.5	0.25	1
Chlorine				**			
	16	8	32	4	0.25	0.12	1
	16	8	32	4	0.5	0.12	1
	32	16	32	4	0.5	0.12	1
Chloramine		*					
	16	32	16	4	0.5	0.12	1
	16	8	16	8	0.5	0.12	1
	32	16	32	8	0.5	0.12	1

^*a*^ No interpretive criteria.

^*b*^ MICs shaded gray are resistant according to the Clinical Laboratory Standards Institute interpretative criteria (27). The wild-type values represent the median of six replicates and the range. The control, chlorine, and chloramine values are the results for each of the biological triplicates. *P < 0.05 and **P < 0.01.

^*c*^ LZD, linezolid; IMI, imipenem; FOX, cefoxitin; AMI, amikacin; CLA (D3); clarithromycin at day 3; CLA (D14), clarithromycin at day 14 (inducible resistance).

Whole-genome sequencing was performed on Mabs sweeps across passages 2, 4, and 6 and exposure conditions to identify the emergence of conserved and mixed mutations. Using the *de novo* assembled genome of the wild-type original isolate as the reference, no mutations were identified in the control (passages 4 and 6) or any of the chlorine or chloramine passaged Mabs.

## DISCUSSION

Mabs isolated from Brisbane DWDS was more susceptible to chloramine than chlorine and had MICs 5× and 300× greater, respectively, than the guideline residual concentration values (3 and 0.5 ppm) for DWDS in Australia (28). Mabs greater susceptibility to chloramine contrasts with literature findings where Mabs and NTM have been more frequently isolated from DWDS using chloramine disinfection (14, 29). Disinfectant efficacy studies have also previously shown that chlorine is the more effective disinfectant against NTM compared with chloramine (30). However, the broth microdilution method used to assess susceptibility was not comparable to conditions in DWDS, and disinfectant susceptibility is not the only factor contributing to Mabs isolation from DWDS. Nutrient availability and pH can influence chlorine susceptibility, with water-grown *Mycobacterium avium* being significantly more resistant than medium grown (30). Biofilm formation (31), aggregation (32), and the presence of amoeba and other bacteria (27) have also been implicated in the disinfection resistance of NTM.

Maa DCC5 has been the most frequently isolated Mabs from water in Brisbane (In Press, AJCRRM 2025) and was significantly more resistant to chloramine than the Mam or Maa DCC1 isolates. Greater chloramine resistance in Maa DCC5 could be a possible explanation for its prevalence in Brisbane’s DWDS, which uses a combination of chlorine and chloramine treatment. Significant variation in disinfection resistance has been found between NTM species (33, 34), chloramine susceptibility between strains of *M. avium* (30), and chlorine susceptibility between colony variants of the same strain (34). Disinfectant resistance did not differ between the time points where Mabs was isolated from Brisbane’s DWDS.

There was also no difference in antibiotic resistance identified for Mabs water isolates between the time points. The Mabs water isolates were amikacin and macrolide susceptible. Macrolide susceptibility was expected due to all Maa isolates being DCC5 isolates, which have the T28C variant rendering the *erm(41*) gene responsible for inducible resistance non-functional and the remaining isolates Mam. Four Mam isolates were sensitive to minocycline, and three of the four were also doxycycline sensitive, which is rarely seen in clinical isolates (35). The generalizability of the susceptibility results to Mabs in DWDS is limited by the small sample size in this study.

A Maa DCC5 isolate was passaged in chlorine and chloramine at concentrations less than the wild-type MIC and greater than those seen in DWDS. Exposure to subinhibitory concentrations of chlorine resulted in a greater than 16-fold increase in chlorine MIC after six passages. The rate of growth also increased across the 6 weeks of passaging, suggesting adaptation to grow better at the subinhibitory chlorine concentration. The chlorine resistance level was not maintained after the storage of Mabs, suggesting that the increase in resistance was a temporary stress response rather than a heritable change. This was supported by whole-genome sequencing not detecting any genetic changes. Previous studies have not been conducted in mycobacteria; however, these findings are consistent with previous findings in *Pseudomonas* spp., where chlorine resistance was due to upregulation of genes involved in the SOS response (36). The classical SOS response is induced when DNA damage is detected; however, there are additional pathways involved in mycobacteria (37), which have not been well characterized in Mabs. Further studies are required to investigate whether relevant genes in Mabs are regulated upon chlorine and chloramine exposure.

Antibiotic resistance did not emerge with exposure to chlorine. However, the chlorine-passaged Mabs was statistically significantly more susceptible to amikacin. Previous studies in *Pseudomonas* (36) and *Salmonella* spp. (38) have found increased resistance to aminoglycosides to co-occur with the evolution of increased chlorine resistance in contrast to this study in Mabs. Increased resistance to other classes of antibiotics with the evolution of increased chlorine resistance has also been shown in other organisms (39). *Pseudomonas* and *Salmonella* spp. have mechanisms of resistance to aminoglycosides, including increased efflux, expression of antibiotic-modifying enzymes, and decreased permeability thought to be induced by exposure to chlorine (37, 38, 40). Mabs has significantly different cell biology from these organisms, and its known mechanisms of acquired antibiotic resistance are point mutations, which may not be as easily induced by disinfectant exposure.

Chloramine-passaged Mabs was statistically significantly less susceptible to imipenem without any change in chloramine MIC or genomic signatures. One of the chloramine-passaged lines exhibited twofold greater imipenem MIC than the range of the wild type. Chloramine exposure may have reduced susceptibility to imipenem for one of the replicates, or the increased MIC may be due to measurement variability. The accepted variability in MIC is within one dilution of the mode (26), which was consistent within the range of the wild type. Measurement of imipenem MIC for NTM by broth microdilution has been found to be the most variable (41). The lack of change in chloramine susceptibility during exposure to chloramine may be because the isolate used was DCC5, which was already less susceptible to chloramine. Moreover, the experiment length and chloramine concentration may not have provided the necessary evolutionary pressure for the susceptibility to change, or the method was not sensitive enough to detect small changes.

Horizontal gene transfer (HGT) has also been implicated as a mechanism of increased antibiotic resistance in chlorine-resistant organisms in DWDS (42, 43). Disinfectant exposure is thought to result in stress responses that increase both the permeability of cell membranes and the expulsion of mobile genetic elements from microorganisms (43). The impact of HGT on increased antimicrobial resistance in Mabs in DWDS could not be assessed in this study. Historically, HGT was not thought to be a common mechanism of acquired antimicrobial resistance in mycobacteria. However, recent work has suggested that HGT does occur (10, 44).

The results of this study cannot be generalized to Mabs within DWDS due to the limitations of the methodology; however, they warrant further investigation. Ct values are typically used as a measure of disinfectant efficacy and have been determined for mycobacteria to be in excess of what can be achieved within DWDS (34). The susceptibility values determined in this study are based on broth microdilution as the gold standard antibiotic susceptibility testing (AST) method for NTM (26) and provide a relative measure of susceptibility rather than the concentration needed to control mycobacterial growth in DWDS. Broth media rather than water is likely to impact the susceptibility of Mabs to disinfectants by creating a chlorine demand, as well as previous work in *M. avium* suggests medium-grown isolates were more susceptible than water grown (30). The interpretation of these results is further complicated by the potential presence of viable but not culturable cells (14, 29). Future studies investigating a larger sample of isolates, a longer evolution experiment duration, and growth conditions more closely resembling those in DWDS would further elucidate the evolution of disinfectant resistance in Mabs.

### Conclusions

Mabs isolated from drinking water at three time points across a 15-year period did not have significant differences in antimicrobial susceptibility. There were subspecies and DCC differences in chloramine susceptibility, and most isolates were significantly more susceptible to chloramine than chlorine. Exposure to sub-MIC concentrations of chlorine in a laboratory evolution experiment resulted in increased chlorine resistance after 6 weeks, while it was not a heritable or genetic change. Increased chlorine resistance did not result in increased antibiotic resistance. Exposure to sub-MIC concentrations of chloramine did not result in changed chloramine susceptibility but may have reduced imipenem susceptibility. Future disinfectant susceptibility testing of a wider range of Mabs isolates could help elucidate the significance of subspecies and DCC differences. The evolution of disinfectant resistance within DWDS could be further investigated using a wild-type strain more susceptible to disinfectants or a longer experiment duration to see if resistance could be more readily or heritably induced, as well as employing an experiment method more representative of conditions in DWDS.

## MATERIALS AND METHODS

### Antimicrobial susceptibility testing of water isolates

Mabs was isolated in 2007, 2017–2018, and 2021–2022 from various tap water outlets in Brisbane as described previously (17) and stored as part of separate projects. There were five isolates from 2007 that had undergone whole-genome sequencing and were identified as Maa belonging to DCC5. It was aimed to have five isolates from each time point to compare. The number and characteristics of Mabs isolates are shown in Table 3.

**TABLE 3:** Characteristics of Mabs water isolates

Year isolated	Sample point	Subspecies	Dominant circulating clone	Chlorine and chloramine susceptibility	Antibiotic susceptibility
2007	SP42	*abscessus*	5	Y^*a*^	Y
2007		*abscessus*	5	Y	Y
2007		*abscessus*	5	Y	Y
2017		*abscessus*	5	Y	Y
2017		*abscessus*	5	Y	Y
2017		*abscessus*	5	Y	Y
2018		*massiliense*	3	Y	Y
2021	SP42	*abscessus*	5	Y	Y
		*abscessus*	1	Y	N^*b*^
2021	Hospital 4 Bathroom sink tap	*massiliense*	Non-DCC	Y	Y
2021	Hospital 4 Bathroom sink tap	*massiliense*	Non-DCC	Y	Y
2021	Hospital 4 Bathroom sink tap	*massiliense*	Non-DCC	Y	Y
2022		*massiliense*	Non-DCC	Y	N

^*a*^ Y, susceptibility testing performed.

^*b*^ N, susceptibility testing not performed.

Isolates were revived by incubation on Middlebrook 7H11 agar for a minimum of 7 days at 30°C before subculture on Mueller Hinton + oleic albumin dextrose catalase (OADC) agar for 72 hours of incubation prior to preparation of the bacterial suspension for AST. AST was performed following the Clinical Laboratory Standards Institute guidelines using Sensititre Myco RAPMYCOI AST plates (Thermo Fisher Scientific). Susceptibility to chlorine and chloramine was also performed by broth microdilution with preparation of a 96-well plate containing final concentrations of disinfectant from 0.5 to 256 ppm in a twofold serial dilution.

No statistics were performed on the antibiotic susceptibility testing data as the sample size was not large enough to consider any change other than a change in category (sensitive, intermediate, and resistant) as significant. No interpretive categories exist for chlorine or chloramine; thus, the chlorine and chloramine MICs were log_2_ transformed for statistical analysis. The difference in chlorine and chloramine susceptibility over time was analyzed with a two-way analysis of variance with Dunnett’s multiple comparisons test of the 2007 results with the 2017–2018 and 2021–2022 results. The effect of subspecies on chlorine and chloramine susceptibility was analyzed with a two-way analysis of variance and Šídák’s multiple comparisons test.

### Serial passaging in chlorine and chloramine

**Fig 4:**
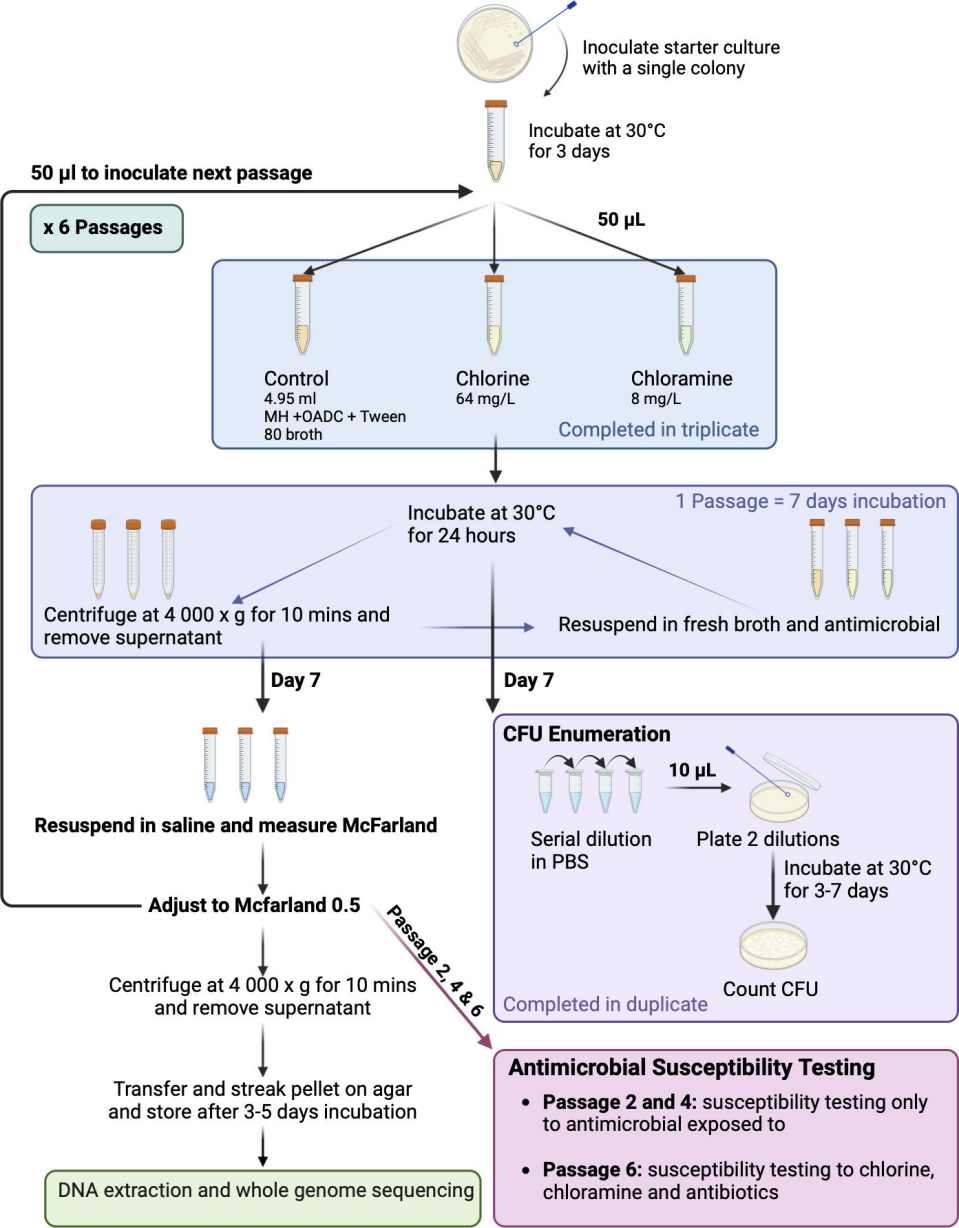
Method used for the evolution of antimicrobial resistance in an *M. abscessus* water isolate. The experiment was completed in biological triplicate. Created with BioRender.com.

The isolate used for the evolution experiment was a 2007 Maa DCC5 isolate from a sample site that had also had Mabs isolated in 2021 and 2022. The method used for the evolution experiment is summarized in Fig. 4. A single colony of the isolate SP42_07 was used to inoculate 2 mL of Mueller Hinton 10% OADC and 0.05% Tween 80 broth as a starter culture. After 3 days of incubation at 30°C to reach 1 × 10^8^ to 1 × 10^9^ CFU/mL, 50 µL was used to inoculate 4.95 mL of broth for four conditions of control, 64 ppm chlorine, and 8 ppm chloramine in triplicate. The concentration of disinfectant used was the highest concentration at which there was still sufficient growth of the Mabs. They were then incubated for 7 days at 30°C for one passage, where the broths were centrifuged at 4,000 × g for 10 min, and the supernatant was removed and resuspended in fresh broth with the relevant disinfectant every 24 hours. Broth and antimicrobial were replaced every 24 hours due to degradation of chlorine and chloramine during incubation. The concentration used for the evolution experiment was determined by a trial of four concentrations from half the MIC to three twofold dilutions lower than the MIC. The highest concentration at which there was sufficient growth after 7 days to form a 0.5 McFarland was chosen.

At the end of each passage (day 7), 50 µL was removed for CFU enumeration prior to centrifugation and removal of supernatant. CFU enumeration was performed in duplicate by serial dilution of 10 µL in PBS to 10^−5^ and 10^−6^ for the control and chloramine lines and 10^−4^ and 10^−5^ for the chlorine. Ten microliter of each dilution was then spread on an agar plate, and colonies were counted after 3–7 days of incubation at 30°C. After centrifugation, the pellets were resuspended in 3 mL of saline and placed in a densitometer to record the McFarland standard. The McFarland standard was then adjusted to 0.5 (~1.5 × 10^8^ CFU/mL), and 50 µL was used to inoculate a fresh 4.95 mL broth with the relevant antimicrobial to commence the next passage. At passages 2, 4, and 6, the 0.5 McFarland standard was also used for AST to the relevant disinfectant as well as antibiotics at passage 6. Susceptibility testing was performed as described above with a change in the range of concentrations as follows. Chlorine and chloramine plates had a twofold serial dilution from 32 to 1,024 ppm as well as 192, 320, 384, and 448 ppm in between. The McFarland suspension was then centrifuged at 4,000 × g for 10 minutes, and the supernatant was removed before the pellet was streaked on Mueller Hinton + OADC agar and incubated for 3–5 days until sufficient growth for storage.

DNA was extracted from the original wild-type isolate as well as from sweeps of stored samples at passages 2, 4, and 6 as described previously (12). DNA was sequenced using Illumina NextSeq (150 bp paired end) at a depth allowing >120 times coverage. Reads were trimmed and quality filtered using Trimmomatic (v0.39-2) (45). *De novo* assembly of the wild-type isolate was performed using SPAdes (v3.15.5) (46) and genes predicted using Prodigal (v.2.6.3) (47). Breseq (v0.38.1) (48) was used to determine genomic variants of passaged cultures, including single-nucleotide mutations, point insertions and deletions, large deletions, and new junctions, using default parameters and the original wild-type isolate as the reference strain. Only assigned, predicted structural variants were investigated.

### Statistics for the evolution experiment

CFU data at each passage were compared to determine if there was a trend over the course of the passage by fitting a mixed model with Tukey’s multiple comparisons test comparing the CFU at each passage with every other passage.

MIC values were log_2_ transformed for all statistical analyses. The significance of the change in MIC over the passages was determined by using a two-way analysis of variance with Šídák’s multiple comparisons test. The significance of changes in MIC to other antimicrobials after passage 6 was determined by a two-way analysis of variance with Dunnett’s multiple comparisons test with every condition compared to the wild-type values.

## Data Availability

Raw sequencing data have been uploaded to the NCBI Sequence Read Archive under accession no. PRJNA1131965.
